# Palliative radiotherapy for gross hematuria in patients with advanced cancer

**DOI:** 10.1038/s41598-021-88952-8

**Published:** 2021-05-05

**Authors:** Mami Ogita, Jiro Kawamori, Hideomi Yamashita, Keiichi Nakagawa

**Affiliations:** 1 Department of Radiology, The University of Tokyo Hospital, 7-3-1 Hongo, Bunkyoku, Tokyo, 113-8655 Japan; 2 Department of Radiation Oncology, St. Luke’s International Hospital, 9-1 Akashicho, Chuoku, Tokyo, 104-8560 Japan

**Keywords:** Oncology, Cancer

## Abstract

This study assessed the efficacy of palliative radiotherapy for gross hematuria caused by advanced cancer. Patients who received palliative radiotherapy to control gross hematuria in two hospitals between October 2006 and May 2020 were retrospectively reviewed. We evaluated the gross hematuria response, gross hematuria control duration, blood transfusion rate, blood transfusion-free duration, and overall survival. Cox multivariate analysis was performed to examine factors associated with hematuria control duration. Fifty-three consecutive patients were included. The most frequently used dose fractionation regimen was 30 Gy in 10 fractions (BED_10_ = 39 Gy), followed by 20 Gy in 5 fractions (BED_10_ = 20 Gy). Forty patients (76%) became gross hematuria free. The median hematuria control duration was 4.3 months (95% confidence interval 1.9–6.6). Twenty-six patients received blood transfusion 3 months before radiotherapy; 17 of them (65%) were free from blood transfusion 1 month after radiotherapy. A high BED_10_ (≥ 36 Gy) was a statistically significant factor for hematuria control duration in the multivariate analysis (*P* = 0.02). Palliative radiotherapy can effectively relieve gross hematuria irrespective of the primary tumor site. A high BED_10_ may be recommended for a prolonged hematuria control duration if patients have a good prognosis.

## Introduction

Cancer patients in advanced stages experience multiple symptoms. Approximately 6–10% of patients with advanced cancer experience clinically significant bleeding^1^. Cancer-related gross hematuria is difficult to control and impairs patient quality of life. Severe hematuria requires frequent blood transfusions and sometimes endangers the patient’s life.

The management and treatment of bleeding depend on the tumor origin and site(s) of bleeding. Urothelial cancer and other cancers directly invading the urinary tract can cause hematuria. The most common cause of cancer-related hematuria is inoperable advanced bladder cancer. There are several methods for the management of vesical bleeding: urethral catheter placement, cystoscopy with laser coagulation and resection of the bleeding site, and endovascular techniques for arterial occlusion^2,3^. Radiotherapy has also been used to controlhematuria for bladder cancer in the palliative setting^4–7^.

Prostate cancer directly invades the urethra or bladder and causes hematuria. Endovascular management is considered for symptom palliation. Palliative radiotherapy, as a minimally invasive procedure, is also used to relieve the bleeding caused by prostate cancer^8^.

Although cancers other than bladder or prostate cancer can cause hematuria due to direct involvement of the urinary tract by the primary tumor or metastases, data on the efficacy of palliative radiotherapy for hematuria caused by various advanced cancers are limited. Therefore, we aimed to assess the effectiveness and safety of palliative radiotherapy on gross hematuria caused by any type of advanced cancer.

## Methods

### Study design and patients

This study was designed as a retrospective cohort study. We retrospectively reviewed all consecutive patients who received palliative radiotherapy between October 2006 and May 2020 in two hospitals, the University of Tokyo Hospital and St. Luke’s International Hospital in Tokyo, Japan, to control gross hematuria. The inclusion criteria were patients who had gross hematuria due to advanced cancer and received radiotherapy with palliative intent. The exclusion criteria were patients who received radiotherapy for definitive treatment or palliative radiotherapy for reasons other than gross hematuria.

### Radiotherapy

All patients underwent CT simulation. Treatment volume and dose fractionation were determined at the discretion of the treating physician. A three-dimensional conformal radiation therapy technique was used for treatment planning. A target dose was prescribed to 100% at the International Commission on Radiation Units and Measurements reference points. Dose calculations were performed with adaptive convolution by the Pinnacle treatment planning system (Philips, Andover, USA). Radiotherapy was delivered with 4–10 MV photon beams in the supine position without specific immobilization. The beam was shaped using a multileaf collimator.

### Data collection

Electronic medical charts were reviewed to obtain data. Data collected included patient demographics such as age, sex, Eastern Cooperative Oncology Group (ECOG) performance status, information on the primary tumor, histology, distant metastasis (present/absent), details of radiotherapy (including the radiotherapy dose, fractionation regimen, and treatment technique), treatment information on chemotherapy and hormonal therapy before and after radiotherapy, blood transfusion records, the status of hematuria, and clinical outcomes. The blood transfusion record was reviewed 3 months before radiotherapy throughout the follow-up period. Adverse events following radiotherapy were collected and graded based on the Common Terminology Criteria for Adverse Events (CTCAE) ver. 4. This study was approved by the Institutional Review Boards of the University of Tokyo Hospital (2020079NI) and St. Luke’s International Hospital (20-R114). All methods were carried out in accordance with relevant guidelines and regulations. According to the guidelines, patients were not required to give informed consent for the present study because this study is retrospective. Instead, we applied the optout method to obtain consent for this study via the web sites of each hospital. Th ethics committees of the University of Tokyo Hospital and St. Luke’s International Hospital waived the need of informed consent and approved the consent procedure.

### Outcome

We evaluated the response to gross hematuria at the end of radiation therapy and 1, 3, 6, and 12 months after radiotherapy. Complete response (CR) was defined as the absence of gross hematuria. Partial response (PR) was defined as an improvement and decrease in the frequency of gross hematuria but not complete disappearance. Overall response (OR) was defined as CR combined with PR. Patients with the same or worse gross hematuria or who required an intervention to stop bleeding, such as endovascular embolization, were considered to have no response (NR). The hematuria control duration was defined as the time from the last day of radiotherapy to the date of the reappearance of gross hematuria or the absence of reappearance of gross hematuria to the date of the last assessment or death. If patients had NR, the hematuria control duration was considered zero. The net gross hematuria control rate was obtained by dividing the period of hematuria control duration by the number of days of survival and multiplying the result by 100. Among the patients who required blood transfusion within 3 months before radiotherapy, the blood transfusion rate and blood transfusion-free duration were calculated from the last day of radiotherapy to the date of retransfusion. Overall survival (OS) was defined as the time from the first day of radiotherapy to death.

### Statistical analysis

Kaplan–Meier analysis was used to calculate OS, the hematuria control duration, and the blood transfusion-free duration. The log-rank test was used to assess the differences. To examine the factors associated with the hematuria control duration, log-rank tests and univariable and multivariable Cox proportional hazards models were used. The covariates included sex, age (< 75 vs. ≥ 75 years), performance status (0, 1 vs. ≥ 2), biologically effective dose_10_ (BED_10_) (< 36 Gy vs. ≥ 36 Gy), blood transfusion within 3 months before radiotherapy, primary tumor site (bladder, upper genitourinary tract, or prostate vs. others), and anticoagulation or platelet treatment. All statistical analyses were performed using SPSS ver. 24 software (IBM Corporation, Armonk, NY, USA).

### Ethics declarations

This study was approved by the Institutional Review Boards of the University of Tokyo Hospital (2020079NI) and St. Luke’s International Hospital (20-R114).

## Results

### Baseline characteristics

In total, 53 consecutive patients who received palliative radiotherapy for gross hematuria between October 2006 and May 2020 were included in this study. The median follow-up duration was 4.9 months (range, 0.2–36). The median patient age was 73 years (range, 36–96). Patient characteristics are shown in Table 1. Twenty-two patients (42%) and sixteen patients (30%) had bladder cancer and prostate cancer, respectively. Forty-seven patients (89%) had distant metastasis.

**Table 1:** Patient characteristics at baseline.

	n	%
**Age, years**		
Median (range)	73	(36–96)
**Sex**		
Male	40	75.5
Female	13	24.5
**Performance status**		
0	1	1.9
1	23	43.4
2	15	28.3
3	8	15.1
4	6	11.3
**Primary tumor**		
Bladder cancer	22	41.5
Prostate cancer	16	30.2
Upper GU tract cancer	5	9.4
Colorectal cancer	5	9.4
Gastric cancer	2	3.8
Esophageal cancer	1	1.9
Cervical cancer	1	1.9
Ovarian cancer	1	1.9
**Histology**		
Urothelial carcinoma	21	39.6
Adenocarcinoma	23	43.4
Squamous cell carcinoma	2	3.8
Others	3	5.7
Unknown	4	7.5
**Presence of distant metastasis**		
Yes	47	88.7
No	4	7.5
Unknown	2	3.8
**Anticoagulation or platelet treatment**		
Yes	6	11.3
No	47	88.7
**Blood transfusion before RT**		
Yes	26	49.1
No	27	50.9
**Chemotherapy**		
Before and/or after RT	22	41.5
Concurrent with RT	3	5.7
Never	28	52.8

Abbreviations:* RT* radiation therapy.

### Radiotherapy

The most frequently used dose fractionation regimen was 30 Gy in 10 fractions (26%), followed by 20 Gy in 5 fractions (23%) and 36 Gy in 12 fractions (21%) (Table 2). Intra-arterial chemotherapy combined with radiotherapy was used in three bladder cancer patients: 50 Gy in 25 fractions in 1 patient, 48 Gy in 24 fractions in 1 patient, and 30 Gy in 10 fractions in 1 patient.

**Table 2:** Radiation dose and schedule.

	BED_10_ (Gy)	n	%
9 Gy/3 fr	11.7	1	1.9
8–10 Gy/1 fr	14.4–20	4	7.5
16 Gy/2 fr, once weekly	24	1	1.9
20 Gy/5 fr	28	12	22.6
20 Gy/4 fr	30	1	1.9
21 Gy/3 fr, every other day	35.7	2	3.8
30 Gy/10 fr	39	14	26.4
36 Gy/12 fr	46.8	11	20.8
35 Gy/10 fr	47.3	2	3.8
40–45 Gy/20–25 fr	48–53.1	2	3.8
48–50 Gy/24–25 fr	57.6–60	3	5.7

Three patients received concurrent intra-arterial chemotherapy with radiotherapy: one 50 Gy in 25 fractions with 5-fluorouracil, one 48 Gy in 24 fractions with cisplatin and pirarubicin, and one 30 Gy in 10 fractions with irinotecan hydrochloride hydrate.

Abbreviations: *fr* fraction.

The target volume for bladder cancer included the whole bladder in 17 patients, bladder tumor sites in 3 patients, and small or whole pelvis in 2 patients. The target volume for prostate cancer included the whole prostate and sites of tumor invasion into the bladder in 9 patients, whole prostate and whole bladder in 3 patients, tumor sites in 3 patients, and prostate in 1 patient. The targets for the other cancers were the tumor sites in 13 patients, tumor sites and whole bladder in 1 patient, and whole bladder in 1 patient.

### Adverse events

Grade 2 adverse events occurred in four patients. Three patients had grade 2 acute diarrhea, and 1 patient had grade 2 acute proctitis. The details of the grade 2 adverse events are shown in Table 3. The patient who experienced grade 2 acute proctitis after palliative radiotherapy (20 Gy in 5 fractions) to the prostate and area of bladder invasion had a history of pelvic irradiation 6 years prior, including 45 Gy in 25 fractions to the whole pelvis plus 26 Gy in 13 fractions to the prostate. Grade 1 adverse events occurred in 7 patients: two acute dermatitis, three acute diarrhea, and two acute genitourinary toxicities. No grade 3 or higher adverse events related to radiotherapy were observed.

**Table 3:** The details of the grade 2 adverse events.

Patient	Grade 2 adverse events	Sex	Age	Primary tumor	Dose fractionation	Concurrent chemotherapy	Treatment volume	Others
1	Acute diarrhea	Male	72	Bladder cancer	50 Gy/25 fr	Intra-arterial chemotherapy with 5-FU	Whole bladder	
2	Acute diarrhea	Male	66	Bladder cancer	48 Gy/24 fr	Intra-arterial chemotherapy with cisplatin and pirarubicin	Small pelvis	
3	Acute diarrhea	Female	39	Ovarian cancer	30 Gy/10 fr	No	The disseminated tumor site of bladder invasion	
4	Acute proctitis	Male	75	Prostate cancer	20 Gy/5 fr	No	Prostate and the tumor site of bladder invasion	History of pelvic irradiation

Abbreviations: *fr* fraction.

### Outcome

**Figure 1:**
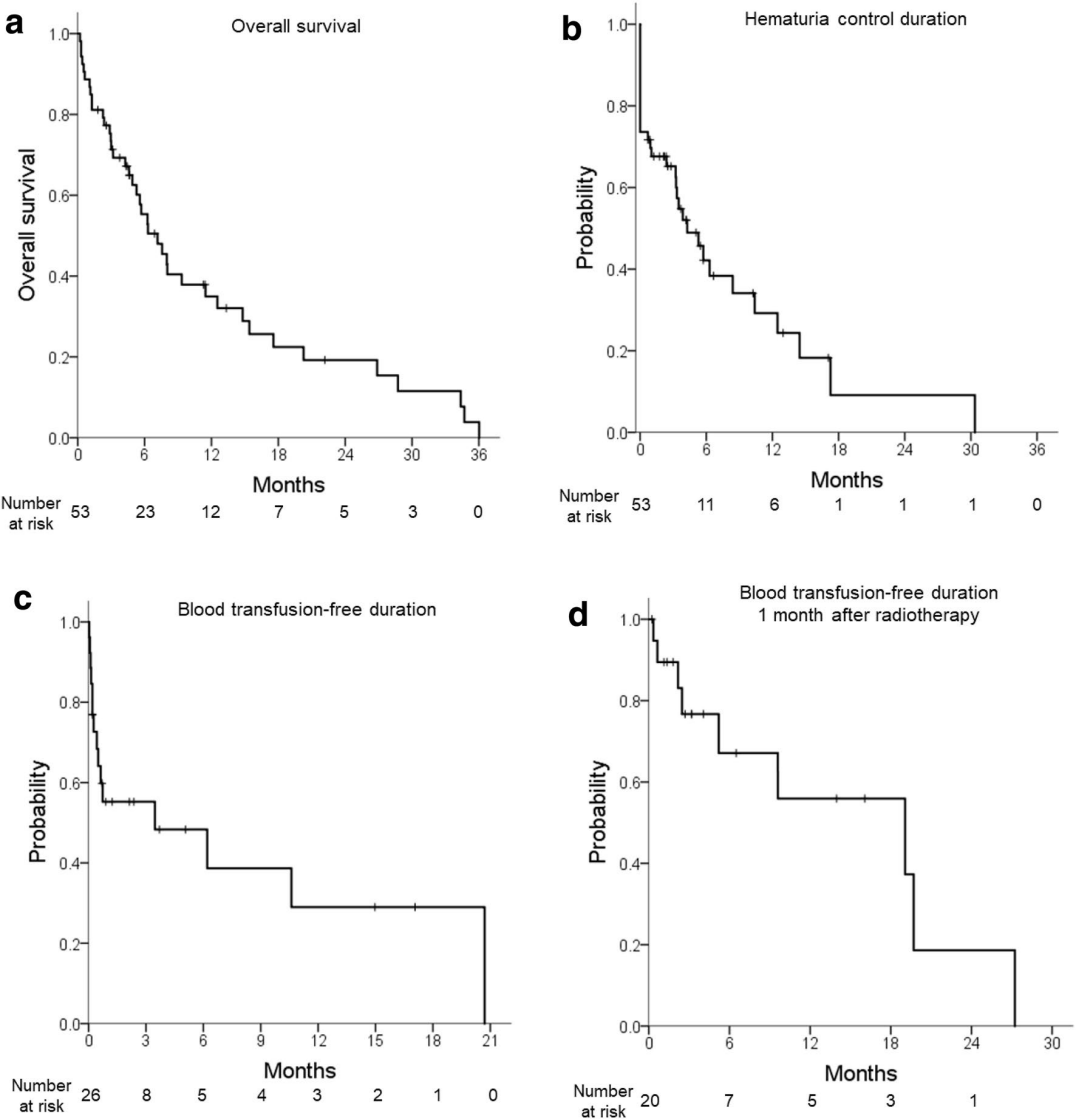
(**a**) Overall survival, (**b**) hematuria control duration, (**c**) blood transfusion-free duration in patients who required blood transfusion within 3 months before radiotherapy, and (**d**) blood transfusion-free duration 1 month after radiotherapy.

OS is shown in Fig. 1a. The 1-year OS rate was 35%. Forty patients died, 36 of whom died of disease progression. Two patients died of other noncancer-related causes. The causes of death in the other two patients were unknown.

The gross hematuria CR and OR (CR + PR) rates were 45% and 59% at the end of radiotherapy and 60% and 64% at 1 month after radiotherapy, respectively (Table 4). In total, 40 and 41 patients achieved CR and OR, respectively. The cumulative CR and OR rates were 76% and 77%, respectively. Among 22 bladder cancer patients, 19 (86%) achieved CR and OR. Among 16 prostate cancer patients, 13 (81%) achieved CR and OR. Among 10 patients who had cancer in a tissue other than the urinary tract or prostate, five (50%) and six (60%) achieved CR and OR, respectively.

**Table 4:** Gross hematuria response rate at the end of radiation therapy and 1, 3, 6, and 12 months after radiation therapy in 53 patients with advanced cancer.

	Complete response		Overall response	
	n	Rate (%)	n	Rate (%)
End of RT	24	45.3	31	58.5
1 month	32	60.4	34	64.2
3 months	23	43.4	25	47.2
6 months	10	18.9	12	22.6
12 months	7	13.2	7	13.2
Cumulative	40	75.5	41	77.4

Complete response (CR) was defined as the absence of gross hematuria. Partial response (PR) was defined as an improvement and a decrease in gross hematuria frequency but not complete disappearance. Overall response (OR) was defined as CR combined with PR.

Abbreviations: *RT* radiation therapy.

The hematuria control duration is shown in Fig. 1b. The median hematuria control duration was 4.3 months (95% confidence interval (CI) 1.9–6.6) in all patients and 8.4 months (95% CI 2.9–13.9) in the patients who were gross hematuria free after radiotherapy. The median net gross hematuria control rate was 56% (range 0–100).

Among 26 patients who received blood transfusion 3 months prior to radiotherapy, 15 (58%) received blood transfusion after radiotherapy. Of them, six patients received blood transfusion only within 1 month after radiotherapy. Seventeen patients (65%) were free from blood transfusions 1 month after radiotherapy throughout the follow-up period. The blood transfusion-free duration is shown in Fig. 1c. The median blood transfusion-free duration was 3.6 months (range 0–10.6). As six patients received blood transfusion only within 1 month after radiotherapy and the maximum response was obtained at 1 month, the blood transfusion-free duration 1 month after radiotherapy completion was also calculated in 20 patients who survived 1 month after radiotherapy (Fig. 1d). The median blood transfusion-free duration in 20 patients was 19.1 months (range 1.0–37.1).

### Prognostic factors for a response

**Figure 2:**
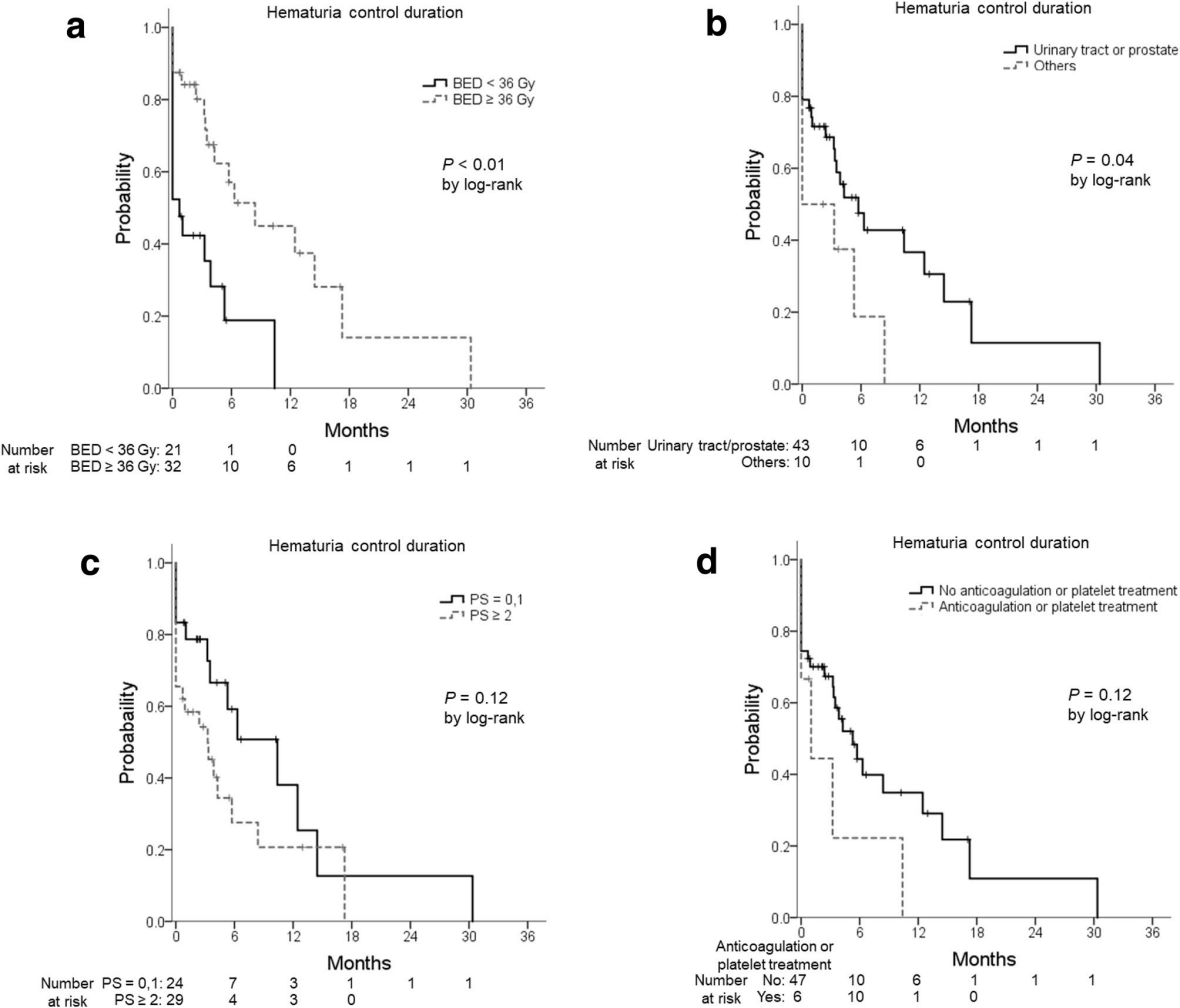
Hematuria control duration (**a**) by the biologically effective dose_10_ (< 36 Gy/≥ 36 Gy), (**b**) by the primary tumor (urinary tract or prostate/others), (**c**) by the performance status (0,1/≥ 2), and (**d**) by anticoagulation or platelet treatment. Abbreviations: *BED* biologically effective dose, *PS* performance status.

In the univariate analysis (log-rank test), BED_10_ ≥ 36 Gy and primary tumor site of the bladder, upper genitourinary tract, or prostate were associated with a prolonged hematuria control duration (*P* < 0.01, *P* = 0.04, respectively). A poor performance status and anticoagulation or platelet treatment were not significantly associated with the hematuria control duration (*P* = 0.12, 0.12, respectively) (Fig. 2). In the multivariate analysis (Cox proportional hazards models), BED_10_ ≥ 36 Gy was a statistically significant factor for a prolonged hematuria control duration (*P* = 0.02, hazard ratio (HR) 0.39, 95% CI 0.18–0.85). The median hematuria control duration was 0.7 months with BED_10_ < 36 Gy versus 8.4 months with BED_10_ ≥ 36 Gy. A performance status ≥ 2 was also associated with a short hematuria control duration, but it was not statistically significant (*P* = 0.06, HR 2.22, 95% CI 0.97–5.08) (Table 5).

**Table 5:** Univariate and multivariate analysis of prognostic factors for the hematuria control duration by Cox proportional hazards models.

	Univariate		Multivariate	
	HR (95% CI)	*P*	HR (95% CI)	*P*
Sex (male/female)	1.42 (0.62–3.25)	0.40		
Age (< 75/≥ 75)	1.09 (0.54–2.23)	0.81		
Performance status (0, 1/≥ 2)	1.70 (0.83–3.49)	0.15	2.22 (0.97–5.08)	0.06
Blood transfusion before RT	1.02 (0.51–2.06)	0.95		
Anticoagulation or platelet treatment	2.01 (0.76–5.31)	0.16	2.65 (0.84–8.41)	0.10
Primary tumor (urinary tract or prostate/others)	2.15 (0.94–4.91)	0.07	1.85 (0.78–4.35)	0.16
BED_10_ (< 36 Gy/≥ 36 Gy)	0.32 (0.15–0.68)	< 0.01	0.39 (0.18–0.85)	0.02

Abbreviations: *HR* hazard ratio, *CI* confidence interval, *RT* radiation therapy, *BED* biologically effective dose.

## Discussion

This retrospective study shows that palliative radiotherapy was associated with a favorable response to gross hematuria caused by advanced cancer irrespective of the primary tumor site. Palliative radiotherapy eliminated gross hematuria in 76% of patients and reduced the demand for blood transfusion according to our results. Adverse events of palliative irradiation were minor, with only grade 2 adverse events in some patients with a history of irradiation or concomitant use of intra-arterial chemotherapy.

Palliative radiotherapy for hematuria caused by bladder cancer is most frequently reported. Intractable hematuria is a common and severe complication in advanced bladder cancer patients^9^. Palliative radiotherapy has been used to relieve bladder cancer-related symptoms since the 1960s^10^, but the reports are limited. Only one randomized controlled trial was conducted to compare the efficacy and toxicity of two hypofractionated radiotherapy regimens (35 Gy in 10 fractions over 2 weeks vs. 21 Gy in 3 fractions on alternate weekdays over 1 week) for the improvement of local symptoms in bladder cancer patients considered unsuitable for curative treatment. Five hundred patients were included in the study, and the data on symptomatic improvements at 3 months were available in 272 patients. In 327 patients with hematuria, including microscopic hematuria, at the start of treatment, 88% experienced symptomatic improvements at 3 months^11^. A phase II study of weekly 6 Gy fractions to a maximum dose of 30–36 Gy in 65 patients was conducted to develop a hypofractionated regimen of bladder palliative radiotherapy for patients with a poor performance status. The results showed that hematuria was controlled in 7/14 patients (50%)^12^. Four retrospective studies evaluated the efficacy of palliative radiotherapy for bladder cancer^4–6,13^. Two studies used a hypofractionated radiation schedule, delivering 30–36 Gy in five to six once-weekly fractions of 6 Gy and 34.5 Gy in six once-weekly fractions of 5.75 Gy. Two other studies used a radiation schedule including 30 Gy in 10 fractions over 2 weeks or 20 Gy in 5 fractions over 1 week and from 40 Gy in 16 fractions to 8 Gy in a single fraction. The rate of hematuria-free status after the completion of radiotherapy was 69–92%, with a median response duration of 3.7–13 months.

A retrospective study showed the results of palliative radiotherapy for hematuria in 25 urothelial carcinoma patients (21 with bladder cancer, 3 with ureter cancer, and 1 with allantoic duct cancer). The rate of complete resolution of macroscopic hematuria was 88%, with a median response duration of 129 days^7^.

Palliative radiotherapy for cancer-related hematuria (other than bladder cancer) has rarely been reported. There are a few reports of palliative radiotherapy for relieving prostate cancer symptoms. Most studies were small retrospective studies conducted before the 1990s^14–20^. Radiotherapy doses varied across studies, ranging from a total dose of 8–70 Gy. The most commonly reported symptoms were obstruction, hematuria, and pain. The hematuria response rate ranged from 42 to 100%. In a systematic review of palliative pelvic radiotherapy for symptomatic, incurable prostate cancer, the pooled hematuria response rate after palliative pelvic radiotherapy was 73%^8^.

In our study, the response rates of specific cancer types to palliative radiotherapy were 86% in bladder cancer patients and 81% in prostate cancer patients. The median response duration was 4.3 months in bladder cancer patients and 12.5 months in prostate cancer patients (data not shown). Our results were comparable to those from previous studies. The response rate in patients with cancers other than those of the urinary tract and prostate was 60%. Those other cancers are the peritoneal dissemination of carcinomas from the various primary sites, and invade the urinary tract wall from the outside. This could make it difficult for radiotherapy to control hematuria. Patients with disseminated disease generally have a more advanced cancer stage than primary urothelial cancer, often with a worse performance. Advanced cancers are usually more aggressive and potentially more radioresistant. Patients with poor performance status do not tolerate the therapy well. These multiple factors could have resulted in the lower response rate. In our study, the primary cancer site of the disseminated disease was not a statistically significant factor for the response. Palliative radiotherapy may beeffective for controlling hematuria regardless of the cancer type, or our study may not have enough power to see the statistical differences.

We chose to analyze the net gross hematuria control rate. This outcome was also used in a previous randomized controlled study of palliative radiotherapy^21^. A longer duration of symptom relief is ideal, but the duration can be largely affected by the life expectancy of the patients in the palliative setting. The net gross hematuria control rate can be minimally affected by the life expectancy of the patients and reflect the efficacy of the treatment.

From our results, BED_10_ ≥ 36 Gy was associated with a longer hematuria control duration than BED_10_ < 36 Gy. A previous study reported that a high BED regimen (> 36 Gy) was associated with a significantly lower rate of recurrent hematuria than a low BED regimen (< 36 Gy) in bladder cancer patients^6^. A randomized trial showed no differences in the response rate for hematuria caused by bladder cancer between two arms: 35 Gy in 10 fractions (BED_10_ = 47.25 Gy) versus 21 Gy in 3 fractions (BED_10_ = 35.7 Gy)^11^. The meta-analysis showed that a higher BED was not associated with improved response rates of hematuria, but a higher BED was associated with a longer duration of hematuria response. There was an associated relative reduction in the hazard of rebleeding of hematuria by 7% for every one Grey increase in BED^22^. The favorable effect of a higher dose in reducing reirradiation was also seen in palliative radiotherapy for lung cancer^23^. Higher doses may greatly reduce the tumor volume and prolong the hemostatic effect. Shorter radiotherapy regimens tend to be used in patients in poor conditions with a poor survival prognosis. Although both the performance status and BED were included in the multivariate analysis, it is possible that there are confounding factors that were not examined. If patients are in poor conditions, a shorter radiotherapy schedule is preferable, but for patients in adequate general conditions and with a satisfactory prognosis, a radiation dose of BED_10_ ≥ 36 Gy may be recommended for a better response and longer hematuria-free duration. It took about one month to observe the response to radiotherapy in this study. Therefore, patients with a life expectancy less than one month may be candidates for the shorter radiotherapy regimen. The objective prognostic tools for advanced cancer patients, such as the Palliative Prognostic Score, the Palliative Prognostic Index, and the Prognosis in Palliative care Study (PiPS) predictor models, may be useful to estimate patient prognosis^24–26^.

To the best of our knowledge, this is the first study to examine the efficacy and safety of palliative radiotherapy for gross hematuria caused by various cancers. Our study has some limitations. First, because of its retrospective nature, the patient characteristics were heterogeneous, and selection bias might have affected the results. Second, the sample size was small, so there was not enough power to detect differences. A prospective study is needed to confirm the effectiveness of a high BED radiotherapy schedule.

Palliative radiotherapy effectively relieved gross hematuria in 76% of patients. A high BED_10_ (≥ 36 Gy) is recommended to improve the hematuria control rate and duration if patients have a good prognosis.

## Data Availability

The datasets used and/or analyzed in the current study are available from the corresponding author on reasonable request.
